# Correction: Fetal cell microchimerism and susceptibility to COVID-19 disease in women

**DOI:** 10.1007/s15010-023-02037-4

**Published:** 2023-05-11

**Authors:** Valentina Cirello, Marina Lugaresi, Alessandro Manzo, Eva Balla, Gerardina Fratianni, Francesca Solari, Luca Persani, Laura Fugazzola, Irene Campi

**Affiliations:** 1 Department of Endocrine and Metabolic Diseases, Istituto Auxologico Italiano IRCCS, Milan, Italy; 2 Department of Pathophysiology and Transplantation, University of Milan, Milan, Italy; 3 Department of Biotechnology and Translational Medicine, University of Milan, Milan, Italy; 4 Department of Cardiovascular Neural and Metabolic Sciences, Istituto Auxologico Italiano, IRCCS, Milan, Italy; 5 Department of Occupational Medicine Unit, Istituto Auxologico Italiano IRCCS, Milan, Italy

**Correction: Infection** 10.1007/s15010-023-02006-x

The Acknowledgements section was missing from this article and should have read "The PhD student Alessandro Manzo was supported by the PhD program in Experimental Medicine of the University of Milan, Milan”.

The original article has been corrected.

